# A Study on the Model of Detecting the Liquid Level of Sealed Containers Based on Kirchhoff Approximation Theory

**DOI:** 10.3390/s17061394

**Published:** 2017-06-15

**Authors:** Bin Zhang, Wen-Ai Song, Yue-Juan Wei, Dong-Song Zhang, Wen-Yi Liu

**Affiliations:** 1 Key Laboratory of Instrumentation Science & Dynamic Measurement, Ministry of Education, North University of China, Taiyuan 030051, China; zb0003@126.com (B.Z.); liuwenyi_nuc@126.com (W.-Y.L.); 2 Science and Technology on Electronic Test & Measurement Laboratory, North University of China, Taiyuan 030051, China; 3 Software School of North University of China, Taiyuan 030051, China; dongsongzhang@hotmail.com

**Keywords:** ultrasonic, sealed containers, liquid level, detecting model

## Abstract

By simulating the sound field of a round piston transducer with the Kirchhoff integral theorem and analyzing the shape of ultrasound beams and propagation characteristics in a metal container wall, this study presents a model for calculating the echo sound pressure by using the Kirchhoff paraxial approximation theory, based on which and according to different ultrasonic impedance between gas and liquid media, a method for detecting the liquid level from outside of sealed containers is proposed. Then, the proposed method is evaluated through two groups of experiments. In the first group, three kinds of liquid media with different ultrasonic impedance are used as detected objects; the echo sound pressure is calculated by using the proposed model under conditions of four sets of different wall thicknesses. The changing characteristics of the echo sound pressure in the entire detection process are analyzed, and the effects of different ultrasonic impedance of liquids on the echo sound pressure are compared. In the second group, taking water as an example, two transducers with different radii are selected to measure the liquid level under four sets of wall thickness. Combining with sound field characteristics, the influence of different size transducers on the pressure calculation and detection resolution are discussed and analyzed. Finally, the experimental results indicate that measurement uncertainly is better than ±5 mm, which meets the industrial inspection requirements.

## 1. Introduction

In the industrial production process, it is important to measure and control the height of liquid media stored in containers for production safety [1,2]. In order to meet different measurement conditions, a variety of liquid level sensors have been developed. At present, there are radar sensors, ultrasonic sensors, radioactive isotope sensors, electronic sensors, thermal liquid level meters, optical liquid level meters, hydraulic pressure gauges, and so on [3].

In some special fields such as petroleum, chemical, and aerospace, the measurement entails more requirements for methods and instruments, especially when a container is to be stored at high temperature or high pressure, and contains inflammable, explosive, highly corrosive, or very volatile liquid inside. In these cases, the detecting sensors cannot be installed in the container directly. An alternative is to use ultrasonic detection technology which can achieve a non-contact and non-immersion measurement without damaging the physical structure and integrity of a container.

Therefore, ultrasonic inspection can be used to determine the liquid level and can provide a guarantee of the safety of the detecting process. Generally, these methods can be classified into four types by the realization principle [1,2,4]. Each method has its own application conditions and limitations [4]. The first type is based on sound speed, in which the liquid level is obtained by measuring the time difference between the emission and reception of waves; the accuracy of these inspections is easily affected by the pressure and temperature in the container. The second type is penetrative methods, in which the liquid level is determined through comparing the attenuation of ultrasonic waves before and after they transmit the gas–liquid medium, but the sound waves can easily be affected by internal impurities and bubbles in the liquid, which may lead to a weak reception. The third type is based on Lamb waves, in which the liquid level is detected through comparing the propagation characteristics; however, these methods require complex initializations and strict conditions, as described in reference [3]. The fourth type is ultrasonic impedance methods, in which the liquid level is determined by comparing the attenuation time of echoes or by comparing the transmission coefficients of sound waves, but the sensitivity is relatively low. In the actual measurement, an appropriate method should be chosen according to the specific requirements of the detection environment.

Given the strengths and weaknesses of these methods, this study presents a model for calculating the echo sound pressure by using the Kirchhoff paraxial approximation theory [5]. Based on this model and according to the different ultrasonic impedance between gas and liquid media, a method for detecting the liquid level from outside of sealed containers is proposed.

The Kirchhoff approximation is a high frequency approximation that allows us to avoid having to solve a boundary value problem in order to determine the far-field scattering amplitude [5,6]. It can be used to deal with all kinds of complex ultrasound field problems; although this theory does not provide an accurate solution, the accuracy of its solution is still higher than that of the analytical solution obtained after a simplified assumption [7]. In ultrasonic nondestructive detection, the Kirchhoff approximation is generally used to simulate the sound field and describe the scattering of flaws or cracks. In this study, the Kirchhoff approximation is introduced to establish a model for calculating echo sound pressure and determining the liquid level for a sealed container.

**Figure 1:**
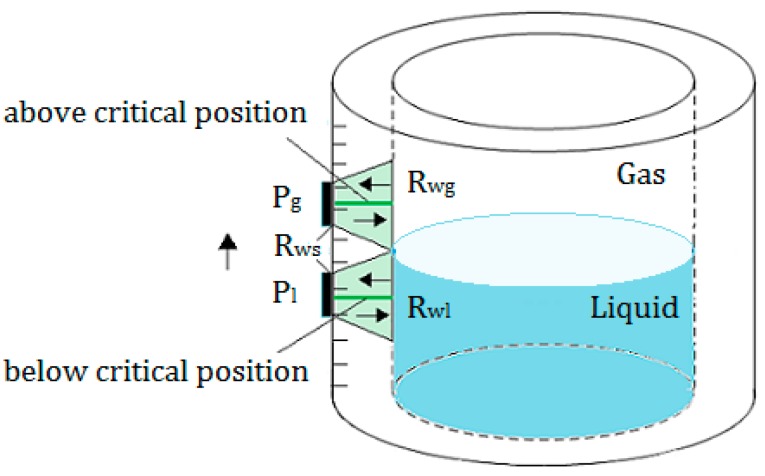
The measurement principle: $R_{\mathrm{wg}}$ represents the reflection coefficient at the inner surface above the liquid level; $R_{\mathrm{wl}}$ is the reflection coefficient below the liquid level. $R_{\mathrm{ws}}$ represents the reflection coefficient at the outer surface of the container wall. $P_{g}$ and $P_{l}$ are the sound pressure relating to the echoes reflected by the inner surface of the container.

As shown in Figure 1, in the actual detection process, in order to find the liquid level, a transducer with trasmitting and receiving functions is moved from the bottom to the top along the outer surface of the container, when the transducer reaches a position below the liquid level. This is because the reflection coefficients $R_{\mathrm{wg}}$ and $R_{\mathrm{wl}}$ at the inner surface of the container wall are not equal. Due to the different impedance between the liquid and gas media in the container, the echo sound pressure detected by the receiving transducer will be changed, while the transducer is continuously moved up to the top until it reaches a position above the liquid level; the echo sound pressure will no longer be changed.

In this process, there are two critical positions above and below the liquid level; between the two positions, the echo sound pressure will be changed from a constant value $P_{g}$ to the other constant $P_{l}$. This study uses the Kirchhoff approximation theory to establish the model for this process and to calculate the echo sound pressure at all the detected positions; through finding the two critical positons, the liquid level can be determined.

## 2. Echo Sound Pressure Calculation Model

### 2.1. Sound Field of a Round Piston Transducer in a Solid

**Figure 2:**
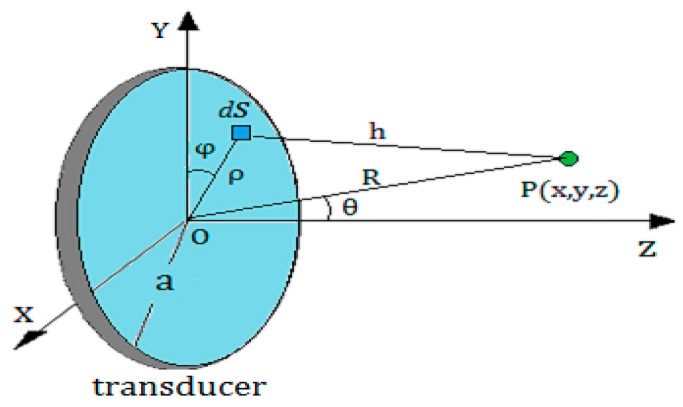
The geometry of the sound field generated by a round piston transducer.

As shown in Figure 2, P(x,y,z) is a point outside the axis of the round piston transducer. The radius of the transducer is a, while the distance from the center of the transducer O(0,0,0) to the point P(x,y,z) is marked by R=D(O,P). The angle between the R and Z-axis is denoted by θ. Then, according to the Kirchhoff integral theorem [8,9,10], the sound pressure amplitude at the point P can be calculated by Equation (1):

(1) $$p\left(R,\theta \right)=\left(\frac{{\pi a}^{2}}{\lambda R}\right)\left[\frac{2J_{1}\left(\mathrm{kasin}\;\theta \right)}{\mathrm{kasin}\;\theta }\right]\cdot p_{0}$$

where $p_{0}$ is the initial sound pressure amplitude of the sound source, λ is the wave length in a medium, k is the wave number; $J_{1}$ is the first order Bessel function; the geometric meaning of other variables is shown in Figure 2.

**Figure 3:**
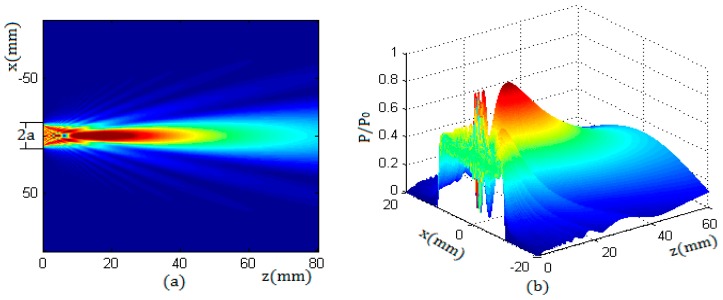
The sound pressure distribution of a round piston transducer in aluminum; (**a**) the 2D distribution in the XOZ plane; and (**b**) the 3D distribution in the YOZ plane.

In Figure 3, the sound field characteristics of a round piston transducer in an aluminum container were simulated by using the Kirchhoff approximation [11,12,13]; the radius of the transducer was 10 mm and the frequency of the transducer was 1 MHz, while in the container wall the compressional wave speed was 6300 m/s and the shear wave speed was 3100 m/s; the ultrasonic impedance of the container was 17 × 10^5^ gm/cm^2^·s.

Figure 3 shows that the sound field of a round piston transducer consisted of two parts: the near field and the far field. In the near field, the sound pressure had many maxima and minima, and in the far field the sound pressure decreased with the increase of distance.

**Figure 4:**
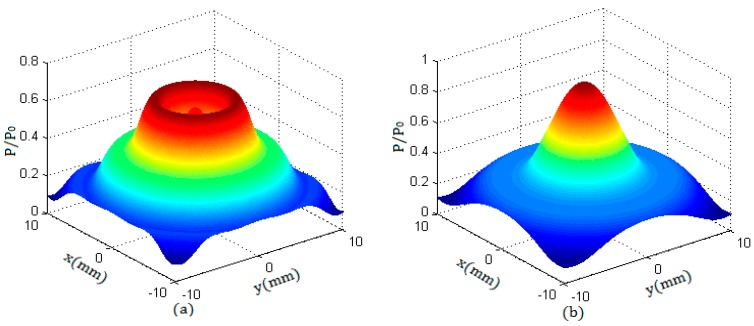

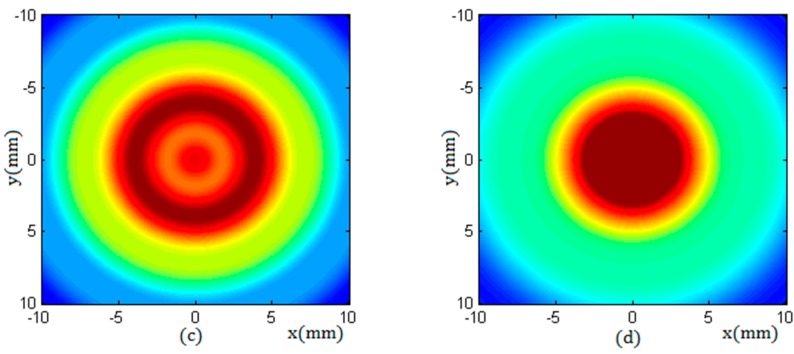
The sound pressure distribution of a round transducer with a radius of 10 mm in cross sections along the beam propagation direction; (**a**,**c**) the beam propagation distance z = 8 mm; (**b**,**d**) the beam propagation distance z = 20 mm.

The sound field of a round transducer is symmetrical along its axis; that is, the sound pressure distribution in any plane passing through the axis is the same as that in the YOZ plane. Therefore, along the propagation direction and in any cross section of the beam, the sound pressure distribution can be obtained. Figure 4 shows the sound pressure distribution of a round transducer with a radius of 10 mm in two cross sections, of which the beam propagation distances were z = 8 mm and z = 20 mm. Due to the fact that the near field length of the transducer N was 15.8 mm, the characteristics in the two sections corresponded to the sound pressure distributions in the near field and in the far field respectively.

From Figure 3 and Figure 4, it can be seen that, in the near field, the beam can maintain a cylindrical shape to transmit. In the far field, the beam propagates with a certain divergence angle. The length of the near field N and the diffusion angle α can be calculated by the equations described in literature [5].

Along the propagating direction of the sound beam, any of the beam cross sections is a circular region; therefore, when a transducer with radius a is used to emit a beam of ultrasonic waves perpendicular to the outer surface of the container wall, at the inner surface, a circle cross section of the beam can be obtained, and its diameter d can be calculated by Equation (2) [4]:

(2) $$\{\begin{array}{c} d=2a\;\left(L\leq N\right)\\ d=2\left[a+\left(L-N\right)\tan\alpha \right]\;(L>N) \end{array}$$

### 2.2. Model of Calculating Echo Sound Pressure

Assuming that the wall thickness of the container is L, the initial incident sound pressure is $P_{0}$ and the average value of the reflected sound pressure at the inner surface of the container wall is expressed by $\bar{p_{r}}$.

**Figure 5:**
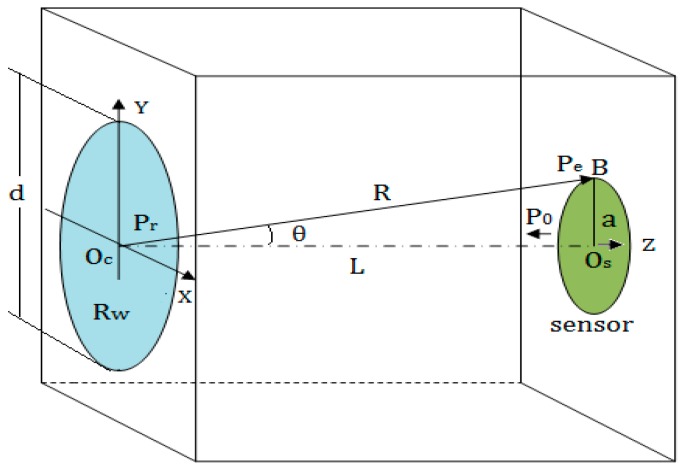
The model for calculating the echo sound pressure by using the Kirchhoff approximation theory.

In Figure 5, the circular section at the inner surface can be approximately regarded as a round transmitting transducer whose average initial pressure is $\bar{p_{r}}$. At any point B on the actual transducer, the sound pressure $p_{e}$ radiated by the circular section can be calculated by Equation (1); furthermore, by integrating the sound pressure $p_{e}$ on the entire surface of the transducer [14,15,16], the average echo sound pressure $\bar{P_{s}}$ can be obtained approximately by Equation (3):

(3) $${\bar{p}}_{s}\left(R,\theta \right)=\left(\frac{\pi {\left(d/2\right)}^{2}}{\lambda R}\right)\left[\frac{2J_{1}\left(k\left(\frac{d}{2}\right)\sin\theta \right)}{k\left(\frac{d}{2}\right)\sin\theta }\right]\cdot \bar{p_{r}}\cdot {\pi a}^{2}$$

where λ is the wave length of ultrasonic waves in a medium, a is the transducer radius, k is the wave number, $J_{1}$ is the first order Bessel function, θ is the angle between R and the Z-axis, $R_{w}$ is the reflection coefficient at the inner surface of the wall, and $\bar{p_{r}}=p_{0}e^{-\alpha L}R_{w}\cdot 4a^{2}/d^{2}$.

**Figure 6:**
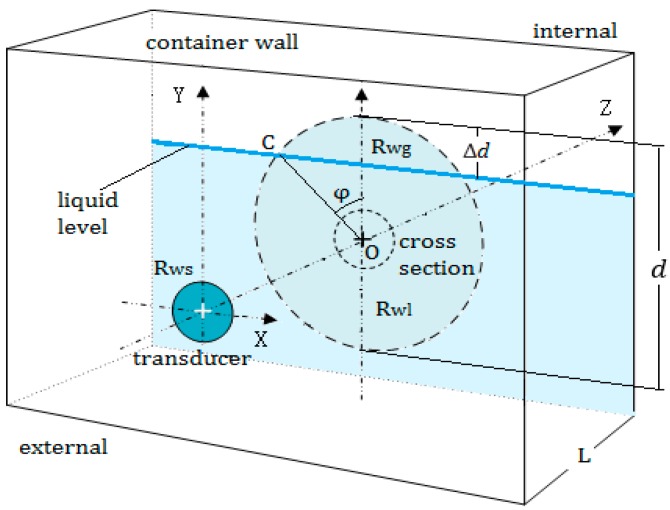
The calculation of the echo sound pressure received by the transducer.${\;R}_{\mathrm{wg}}$ and $R_{\mathrm{wl}}$ represent the reflection coefficients at two parts of the circle section, respectively.

In the actual detection, when the transducer is moved up along the outer surface of the wall and the top of the beam cross section at the inner surface exceeds the liquid level, the exceeding height is represented by Δd and 0≤Δd≤d, as shown in Figure 6.

Assuming that the total area of the circle section is A, the area above the liquid level is expressed by $A_{e}$, and let $r_{s}=A_{e}/A$, the ratio $r_{s}$ can be calculated by Equation (4).

(4) $$r_{s}=\frac{1}{\pi }\left(\varphi -\sin\varphi \cos\varphi \right)$$

where φ is the angle between the line OC and the Y-axis and 0≤φ≤π, C is a cross point of the circle section and the liquid level, O is the center of the circle section.

In the cross section of the beam, the energy is mainly concentrated at the inner surface of the container wall; part of the beam will propagate into the container, the other part of the beam will be reflected because of the discontinuous impedance, which follows the reflection principle of the sound wave.

When 0≤Δd≤d, the circle section is divided into two parts by the liquid level; the echo sound pressure received by the transducer should be calculated by superimposing the two parts of the circle section. It is assumed that the reflected echoes in the wall will decay to a very small amount after n times, which can be neglected relative to the total energy received by the receiving transducer.

Therefore, when the sound beam was reflected to the outer surface of the wall after n times, the total echo sound pressure of the transducer $\sum^{\text{​}}p_{s}$ can be derived as the following equation:

(5) $$\begin{array}{l} \sum^{\text{​}}p_{s}\left(R,\theta \right)=\left(\frac{\pi {\left(d/2\right)}^{2}}{\lambda R}\right)\left[\frac{2J_{1}\left(k\left(\frac{d}{2}\right)\sin\theta \right)}{k\left(d/2\right)\sin\theta }\right]\cdot P_{0}\cdot {\pi a}^{2}\cdot {\left(\frac{2a}{d}\right)}^{2}\\ \;\cdot \left(r_{s}\cdot \overset{n}{\underset{i=1}{\sum }}R_{\mathrm{wg}}^{i}R_{\mathrm{ws}}^{i-1}e^{-\left(2i-1\right)\alpha L}+(1-r_{s})\cdot \overset{n}{\underset{i=1}{\sum }}R_{\mathrm{wl}}^{i}R_{\mathrm{ws}}^{i-1}e^{-\left(2i-1\right)\alpha L}\right) \end{array}$$

where α is the attenuation coefficient of the ultrasonic waves in the container, L is the wall thickness, $R_{\mathrm{wg}}$, $R_{\mathrm{wl}}$ and $R_{\mathrm{ws}}$ are the reflection coefficients.

The Kirchhoff approximation theory is established on the paraxial approximation, and should satisfy the condition z/a >> 1, where a is the transducer radius, and z is the axial distance from the center of the transducer. In practice, in the far field, or even in the outer region of half of the near field, this condition can be satisfied. Therefore, the model based on the Kirchhoff approximation can accurately describe the sound field distribution in the far field and in the outer region of half of the near field. Generally, in the actual detection, the object to be detected should be placed in the far field, so that the calculation error is limited to a small range.

## 3. Experimental Results

### 3.1. Experimental Setup and Initial Conditions

**Figure 7:**
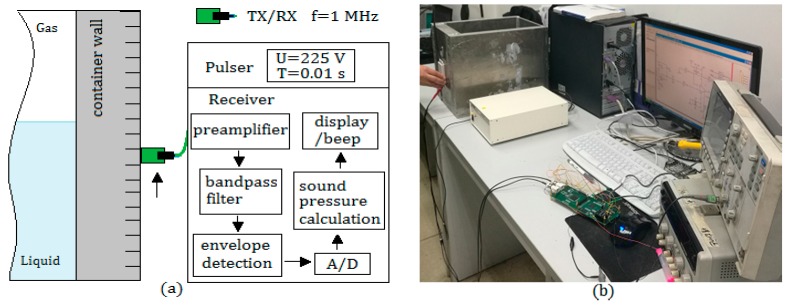
Measurement system: (**a**) TX/RX is a transducer with transmitting and receiving function; and (**b**) calibration devices in the experiments.

The experimental setup of the detection system and calibration devices are shown in Figure 7. In the evaluation experiments of the proposed method, an aluminum container with four different wall thicknesses was used; three kinds of liquid media were selected as detected objects in the experiments—water, edible oil and glycerin—and the gas medium was air in the container.

Table 1 shows the system parameters and some initial values used in the experiments. Water was used as an ultrasonic couplant and the environment temperature was 10~60 ℃.

### 3.2. The Result of Model Simulation

**Figure 8:**
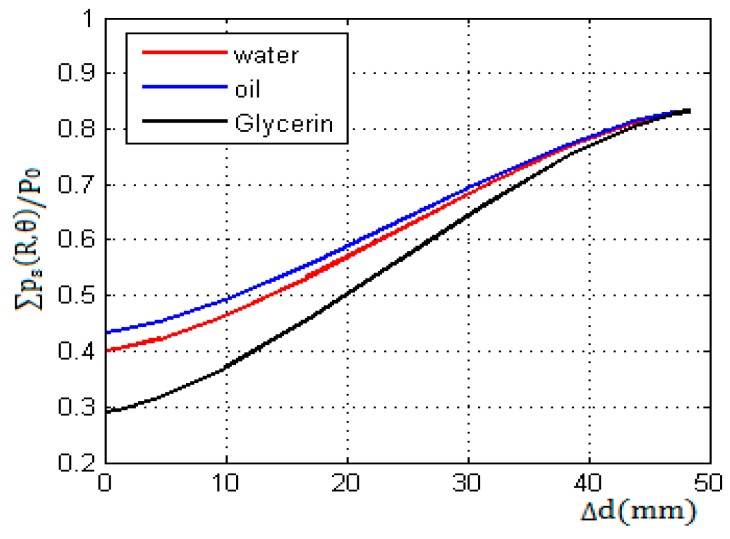
The echo sound pressure versus the exceeding height above the liquid level. The wall thickness L = 50 mm; the abscissa axis is the exceeding height Δd, as defined in Figure 6.

Using the above initial conditions and Equation (5) in the calculating model, the echo sound pressure near the liquid level is calculated and simulated in MATLAB. The results after normalization are shown in Figure 8.

Figure 8 shows the changing characteristics of the echo sound pressure in simulation when the exceeding height Δd increased from 0 to d with the moving of the transducer.

### 3.3. Calculation of Echo Sound Pressure

**Figure 9:**
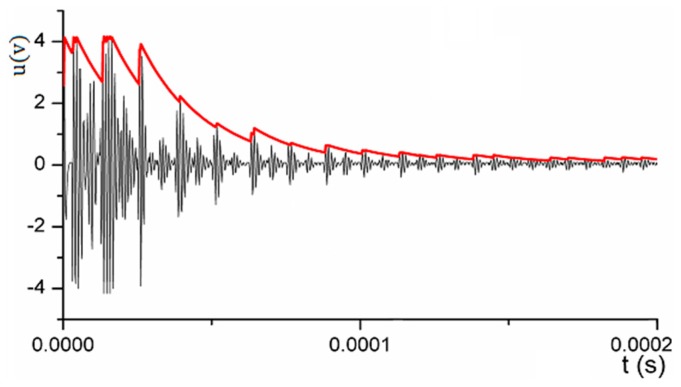
Original echo signal with a 10 MHz sampling and the envelope detection curve.

In Figure 9, the waveform was obtained by sampling the original echo waves at a frequency of 10 MHz; the red line was the envelope detection curve, by which the reflected times of echoes can be determined. The envelope detection curve can be expressed by a function $y_{t}=U\left(t\right)$; as long as the interval between the two transmitting pulses is large enough, the echo energy always decreases from a maximum to zero. Therefore, in a reception period, the envelope detection function $y_{t}=U\left(t\right)$ converges to zero; this characteristic can be used to calculate the reflected times n in Equation (5).

Assuming that the ultrasound wave speed was $v_{m}$, the repetition period T of the transmitting pulse was divided into m segments; each segment was Δt=T/m and $2L/v_{m}<\Delta t<4L/v_{m}$. Let $E_{i}=\overset{i\Delta t}{\underset{\left(i-1\right)\Delta t}{\sum }}\left|y_{t}\right|$ and $\Delta E_{i}=E_{i+1}-E_{i}\;(0<i\leq m)$; when $\lim\Delta E_{i}\to 0$, the reflection times n can be calculated by $n=i\Delta t/\left(2L/v_{m}\right)$. That is, after n times reflection, the echo energy attenuated to a very small amount which can be neglected.

**Figure 10:**
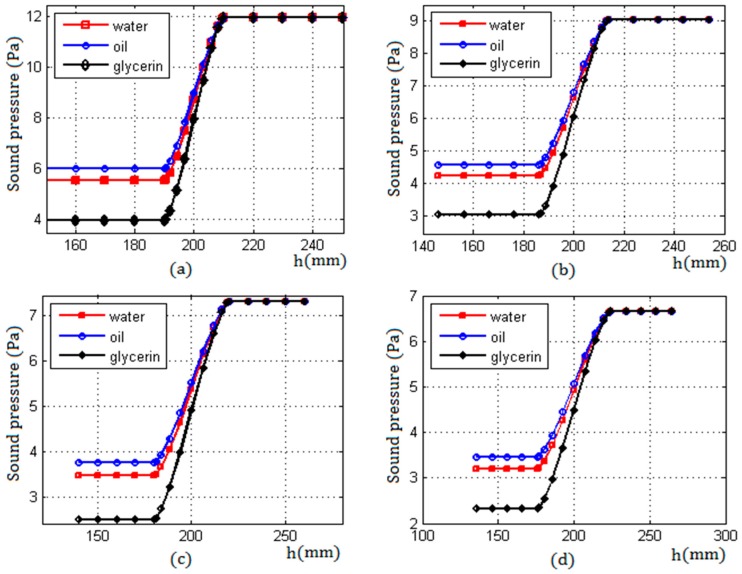
The echo sound pressure of three kinds of liquid media with different ultrasonic impedance calculated under different wall thicknesses; the abscissa axis h represents the scale value of the container height. (**a**) L = 8 mm; (**b**) L = 25 mm; (**c**) L = 40 mm; and (**d**) L = 50 mm.

When n was determined, and according to the parameters in Table 1, the echo sound pressure received by a transducer with a radius of 20 mm, at different detected positions, can be calculated by Equation (5). Figure 10 shows the curve characteristics of the echo sound pressure of three liquid media with different ultrasonic impedance under four sets of different wall thicknesses. Comparing the results shown in Figure 8 and Figure 10, it can be seen that the change law of the echo sound pressure obtained by the model is consistent with the curve of the echo sound pressure in the simulation near the liquid level.

From Figure 10, it can be seen that, for three different ultrasonic impedance liquids, the characteristics of the echo sound pressure were similar in the entire detection process. When the transducer was moved below the liquid level, the echo sound pressure was a smaller constant which was expressed by $P_{\min}$; when the transducer was moved up continuously through the two critical positions, the echo sound pressure increased from a smaller constant $P_{\min}$ to a bigger constant $P_{\max}$; and when the transducer was moved above the liquid level, the echo sound pressure maintained a constant $P_{\max}$ and no longer changed.

The difference of the echo sound pressure between the two critical positions was denoted by $\Delta P=P_{\max}-P_{\min}$. For three liquid media with different ultrasonic impedance, Figure 10 shows that the echo sound pressure difference ΔP was smallest when the liquid in the container was edible oil with the smallest ultrasonic impedance; and when the liquid was glycerin with the biggest ultrasonic impedance, the echo sound pressure difference ΔP was also the biggest. Therefore, the bigger the ultrasonic impedance of the liquid medium, the bigger the difference of the echo sound pressure between the two critical positions, and vice versa.

In addition, it can also be seen that the echo sound pressure was affected by the container wall thickness L; when initial detection conditions were unvaried, the larger the wall thickness of the container, the greater the attenuation of sound waves, and the smaller the echo sound pressure.

From the above analysis, it can be deduced that, in the proposed model for detecting the liquid level, there were two critical factors that influenced the echo sound pressure: one was the ultrasonic impedance of the liquid medium in the container, and the other was the attenuation of ultrasound waves associated with the material and wall thickness of the container. Since the liquid level was determined by the characteristics of the echo sound pressure between the two critical positions in the proposed model, the greater the difference between the two critical values, the higher the resolution, and the higher the detection accuracy, and vice versa.

### 3.4. Determining the Liquid Level by Echo Sound Pressure

**Figure 11:**
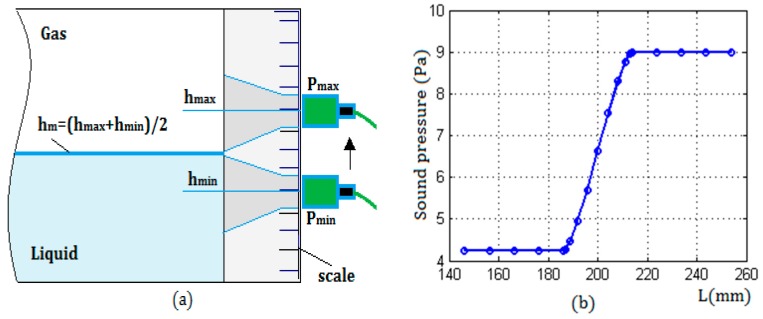
(**a**) The method for determining the liquid level: $P_{\max}$ and $P_{\min}$ were the echo sound pressure corresponding to the two critical positions respectively; $h_{\max}$ and $h_{\min}$ were scale values associated with $P_{\max}$ and $P_{\min}$; $h_{m}$ is the height of the measured liquid level; and (**b**) is a sample of the changing characteristics of the echo sound pressure measured by using a transducer with the radius a = 10 mm, the wall thickness L = 25 mm, and the liquid was water.

Figure 11a shows a schematic diagram of the detection method for determining the liquid level. Figure 11b shows the changing characteristics of the echo sound pressure. From them, it can be seen that the two critical positions can be determined by finding the maximum and minimum of the sound pressure, and according to the analysis of the detection model, the position of the liquid level was the midpoint of the two critical positions. In the detection process, the two critical positions can be obtained by the scale of the container, or can be measured by using an infrared distance measuring device.

### 3.5. Results of Experiment

In this experiment, taking water as an example, the height of the actual liquid level was 200 mm; two kinds of transducers were used to measure the liquid level under four sets of different wall thicknesses, respectively.

Table 2 shows the experimental results; $P_{\max}$ and $P_{\min}$ were the echo sound pressure corresponding to the two critical positions respectively; $h_{m}$ was the height of the measured liquid level, and all the measured data were the average values of three time measurements.

**Figure 12:**
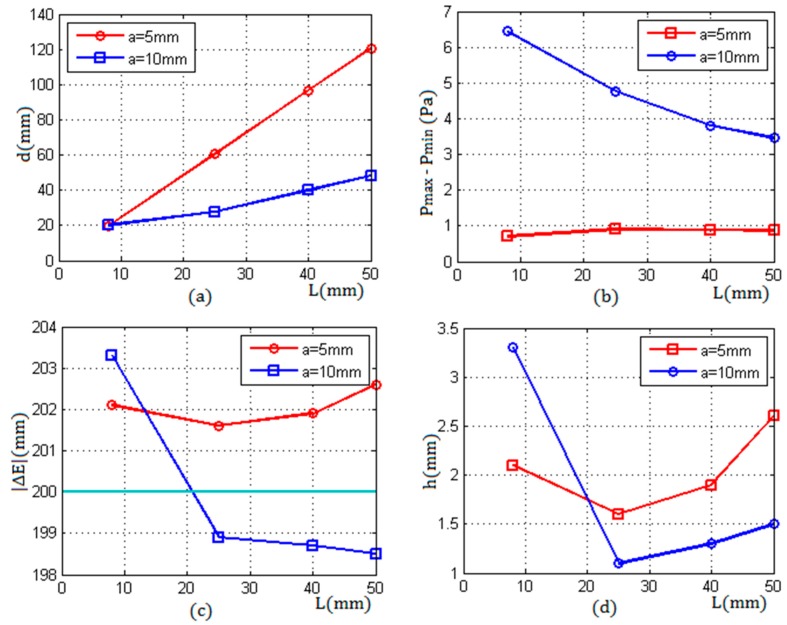
The experimental results taking water as an example: (**a**) the diameters of the transmitting beam cross section versus the wall thickness; (**b**) the difference of the echo sound pressure between the critical positions versus the wall thickness; (**c**) the measured liquid level versus the wall thickness; (**d**) the errors versus the wall thickness.

From Figure 12a, it can be seen that, with the increase of the propagation distance in the container wall, the beam transmitted by the transducer with a radius of 5 mm diverged faster than that emitted by the transducer with a radius of 10 mm. For the same wall thickness, the diameter of the cross section at the inner surface produced by the 5 mm transducer was about 2~3 times bigger than that produced by the 10 mm transducer. Correspondingly, the bigger the d was, the weaker the average sound pressure in the cross section was, and the lower the detection resolution was.

Figure 12b shows the difference of the echo sound pressure between the critical positions versus the wall thickness L; the difference ΔP was measured by two transducers with radii of 5 mm and 10 mm respectively. From Figure 12b, it can be seen that, when using the transducer with a radius of 5 mm, the difference under different wall thicknesses was about 1 Pa, and had a little change. When the radius was 10 mm, with the increase of the wall thickness, the difference of the echo pressure decreased gradually; compared with the radius of 5 mm, the difference ΔP increased obviously, which was beneficial to improve the detection resolution.

Figure 12c shows the comparison between the actual liquid level and the measuring results that were obtained by using two transducers with different radii. The results measured under different wall thicknesses were higher than the actual value of the liquid level when using the transducer with a radius of 5 mm. Conversely, the results were lower than the actual liquid level when measured by the transducer with a radius of 10 mm, except for the wall thickness L = 8 mm; in this case, the thickness was less than the length of the near field N = 15.87 mm, which resulted in a bigger error.

Figure 12d shows the measurement errors associated with two different transducers versus four sets of wall thickness, respectively. From Figure 12d, it can be seen that, when the wall thickness L = 8 mm, the measurement error of the transducer with a 5 mm radius was less than that of the transducer with a 10 mm radius. When the wall thickness L ≥ 25 mm, the errors of the transducer with a radius of 10 mm were less than the errors of the transducer with a 5 mm radius. In the case of the wall thickness L = 25 mm, both of the measurement errors of two different transducers were minimum values respectively.

## 4. Discussion

Through the analysis of the model and experiments, it can be deduced that there are two main factors that affect the measurement accuracy.

On the one hand, the liquid level is determined by the echo sound pressure difference which is associated with the ultrasonic impedance of the liquid and gas media in containers. Experimental results show that, for a liquid medium with a bigger ultrasonic impedance, the difference of the echo sound pressure between two critical positions is more obvious than that for a liquid with a smaller ultrasonic impedance. Therefore, the characteristics of the echo sound pressure depend on the ultrasonic impedance of the liquid media in containers; for a liquid with a very small ultrasonic impedance, the detection resolution will be reduced, or the proposed method is no longer applicable.

On the other hand, the detection model established in this study is based on the Kirchhoff paraxial approximation theory. In actual measurement, to improve the measurement accuracy, the length of the container wall thickness should be placed in the far field of the detection sound field; in order to meet this requirement, a transducer of appropriate size should be chosen according to the actual detection conditions, because the size of the cross section at the inner surface and the average sound pressure in the section are mainly determined by the size of the transducer.

Furthermore, in theory, with the inside pressure increase, the measurement resolution of the method will be reduced. However, since the ultrasonic impedance difference between the gas and liquid media is quite big, for the proposed method, the influence of the container’s inside pressure on the measurement can generally be neglected.

## 5. Conclusions

In this study, the Kirchhoff approximation theory is introduced to conduct liquid level detection of sealed containers, based on which, the model for calculating the echo sound pressure is established. Through the simulation in MATLAB, the correctness and feasibility of the theoretical model are verified, and from experimental results, under a static measurement condition, the measurement accuracy of the model is less than ±5 mm for many common liquids or mixed liquids in industry.

According to the experimental results, it is recommended to use a smaller transducer with a radius less than 5 mm when the wall thickness is less than 25 mm; on the contrary, it is recommended to use a larger transducer with a radius of more than 10 mm when the wall thickness is more than 25 mm.

Furthermore, to improve the stability and reliability, more than one transducer could be used in the same measurement, and the average value of the multiple results could be taken as a final result of the measurement.

Compared with other ultrasonic methods for detecting the liquid level, in the proposed model, variables and parameters in the detection sound field can be easily determined, by which, the reflected echo sound pressure can be calculated effectively and quickly. Therefore, the proposed model reduces the calculation difficulty, improves the detection efficiency, and avoids the limitation of mathematical analysis methods in calculating the complex sound field.

## Figures and Tables

**Table 1:** Experimental parameters and initial values.

Liquid	ρ (g/cm^3^)	Zl (gm/cm^2^·s)	Zg (gm/cm^2^·s)	Zm (gm/cm^2^·s)	Rwg	Rwl	Rws
water	1	1.48 × 10^5^	0.0004 × 10^5^	17 × 10^5^	0.999	0.839	0.839
edible oil	0.91	1.28 × 10^5^	0.0004 × 10^5^	17 × 10^5^	0.999	0.859	0.839
glycerin	1.27	2.42 × 10^5^	0.0004 × 10^5^	17 × 10^5^	0.999	0.750	0.839

**Table 2:** Experimental results.

a (mm)	f (MHz)	L (mm)	N (mm)	d (mm)	P_min_ (Pa)	P_max_ (Pa)	$\bar{h_{m}}$ (mm)	$\left|\bar{\Delta E}\right|$ (mm)
5	1	8	3.96	19.68	0.61	1.31	202.1	2.1
5	1	25	3.96	60.53	0.81	1.72	201.6	1.6
5	1	40	3.96	96.58	0.81	1.69	201.9	1.9
5	1	50	3.96	120.60	0.79	1.66	202.6	2.6
10	1	8	15.87	20	5.50	11.95	203.3	3.3
10	1	25	15.87	27.59	4.22	8.99	198.9	1.1
10	1	40	15.87	40.08	3.48	7.29	198.8	1.2
10	1	50	15.87	48.41	3.20	6.66	198.5	1.5
